# Evaluation of culturally sensitive educational video for informed screening decisions among migrants

**DOI:** 10.1093/eurpub/ckac130.142

**Published:** 2022-10-25

**Authors:** N Hamdiui, ML Stein, JE van Steenbergen, R Crutzen, M Bouman, A Khan, MN Çetin, A Timen, METC van den Muijsenbergh

**Affiliations:** 1 Centre for Infectious Disease Control, RIVM, Bilthoven, Netherlands; 2 Department of Primary and Community Care, Radboud University Medical Center, Nijmegen, Netherlands; 3 Centre for Infectious Diseases, Leiden University Medical Centre, Leiden, Netherlands; 4 Department of Health Promotion, Maastricht University, Maastricht, Netherlands; 5 Center for Media and Health, Gouda, Netherlands; 6 Athena Institute, VU University Amsterdam, Amsterdam, Netherlands; 7 Program Prevention and Care, Pharos, Utrecht, Netherlands

## Abstract

**Background:**

In the Netherlands, especially Turkish- and Moroccan-Dutch women show low cervical cancer screening participation and limited informed decision-making in this regard. To meet the needs of these women, a culturally sensitive educational video was developed. The objective was to evaluate the effect of the video on informed decision-making regarding cervical cancer screening participation among Turkish and Moroccan women aged 30-60 years in the Netherlands.

**Methods:**

Initial respondents were recruited via several social media platforms and invited to complete an online questionnaire. Following respondent-driven sampling, respondents were asked to recruit a number of peers from their social network to complete the same questionnaire. Respondents were randomly assigned to the control condition (current information brochure) or intervention condition (brochure and video). We evaluated the added effect of the video on knowledge, attitude, intention, and informed decision-making using intention-to-treat analyses.

**Results:**

The final sample included 686 Turkish- and 878 Moroccan-Dutch women. Of this sample, 793 were randomised to the control group (350 Turkish and 443 Moroccan) and 771 to the intervention group (336 Turkish and 435 Moroccan). Among Turkish-Dutch women, 33.1% of the control respondents and 40.5% of the intervention respondents consulted the brochure (not statistically significant). Among Moroccan-Dutch women, these percentages were 28.2% and 37.9%, respectively (P = 0.003). Of all intervention respondents, 96.1% (Turkish) and 84.4% (Moroccan) consulted the video. The video resulted in more positive screening attitudes among Moroccan-Dutch women, in comparison to the brochure (74.3% versus 68.4%, P = 0.07).

**Conclusions:**

Our short, easily implementable video resulted in more positive screening attitudes in Turkish- and Moroccan-Dutch women and can thus contribute to informed cervical cancer screening decisions.

**Key messages:**

- Audio-visual interventions lead to a greater reach and impact in informed decision-making among immigrant women, compared to the currently implemented textual information materials.
- Culturally sensitive educational videos, tailored to the needs of vulnerable populations, contribute to informed decision-making and participation in European preventive healthcare programmes.

